# Supplementary material for: Association between an inflammatory biomarker score and future dementia diagnosis in the population-based UK Biobank cohort of 500,000 people
Source: PLoS One. 2023 Jul 19;18(7):e0288045. doi: 10.1371/journal.pone.0288045 (PMC10355406; doi:10.1371/journal.pone.0288045)
Supplement: S1 Table — (DOCX) [file pone.0288045.s001.docx]

| Predictors | OR | Coefficient | p-value | 95% CI lower | 95% CI upper |
| --- | --- | --- | --- | --- | --- |
| **Prospective memory (UKB Field Code 20018)** | | | | | |
| 1st quartile | Reference |  |  |  |  |
| 2nd quartile | 1.076 |  | p<0.001 | 1.036 | 1.119 |
| 3rd quartile | 1.105 |  | p<0.001 | 1.064 | 1.147 |
| 4th quartile | 1.204 |  | p<0.001 | 1.160 | 1.250 |
| Sex | 0.919 |  | p<0.001 | 0.895 | 0.943 |
| Age | 1.043 |  | p<0.001 | 1.041 | 1.044 |
| *APOE* | 0.979 |  | 0.152 | 0.950 | 1.008 |
| Cardiovascular problems | 1.106 |  | p<0.001 | 1.074 | 1.138 |
| Ethnicity | 2.520 |  | p<0.001 | 2.438 | 2.605 |
| TDI | 1.066 |  | p<0.001 | 1.062 | 1.071 |
| **Verbal and numerical reasoning (Fluid intelligence, UKB Field Code 20016)** | | | | | |
| 1st quartile | Reference |  |  |  |  |
| 2nd quartile |  | -0.125 | p<0.001 | -0.156 | -0.093 |
| 3rd quartile |  | -0.229 | p<0.001 | -0.261 | -0.197 |
| 4th quartile |  | -0.366 | p<0.001 | -0.398 | -0.334 |
| Sex |  | 0.246 | p<0.001 | 0.223 | 0.268 |
| Age |  | -0.020 | p<0.001 | -0.022 | -0.019 |
| *APOE* |  | 0.002 | 0.855 | -0.023 | 0.027 |
| Cardiovascular problems |  | -0.192 | p<0.001 | -0.218 | -0.167 |
| Ethnicity |  | -1.123 | p<0.001 | -1.157 | -1.088 |
| TDI |  | -0.074 | p<0.001 | -0.078 | -0.070 |
| **Processing speed (Reaction time, UKB Field Code 20023)** | | | | | |
| 1st quartile | Reference |  |  |  |  |
| 2nd quartile |  | 2.533 | p<0.001 | 1.589 | 3.477 |
| 3rd quartile |  | 4.227 | p<0.001 | 3.279 | 5.175 |
| 4th quartile |  | 8.819 | p<0.001 | 7.860 | 9.777 |
| Sex |  | -18.897 | p<0.001 | -19.570 | -18.225 |
| Age |  | 4.191 | p<0.001 | 4.148 | 4.234 |
| *APOE* |  | 0.726 | 0.059 | -0.027 | 1.480 |
| Cardiovascular problems |  | 5.423 | p<0.001 | 4.656 | 6.191 |
| Ethnicity |  | 45.675 | p<0.001 | 44.391 | 46.960 |
| TDI |  | 2.780 | p<0.001 | 2.668 | 2.893 |
| **Visual declarative memory (Pairs matching, UKB Field Code 399)** | | | | | |
| 1st quartile | Reference |  |  |  |  |
| 2nd quartile |  | -0.032 | 0.027 | -0.061 | -0.004 |
| 3rd quartile |  | -0.055 | p<0.001 | -0.084 | -0.027 |
| 4th quartile |  | -0.096 | p<0.001 | -0.125 | -0.067 |
| Sex |  | -0.048 | p<0.001 | -0.068 | -0.027 |
| Age |  | 0.060 | p<0.001 | 0.059 | 0.061 |
| *APOE* |  | 0.028 | 0.015 | 0.006 | 0.051 |
| Cardiovascular problems |  | 0.040 | 0.001 | 0.017 | 0.063 |
| Ethnicity |  | 0.758 | p<0.001 | 0.720 | 0.796 |
| TDI |  | 0.026 | p<0.001 | 0.022 | 0.029 |
| **Working memory (Numeric memory, UKB Field Code 4282)** | | | | | |
| 1st quartile | Reference |  |  |  |  |
| 2nd quartile |  | -0.062 | 0.001 | -0.098 | -0.026 |
| 3rd quartile |  | -0.104 | p<0.001 | -0.140 | -0.069 |
| 4th quartile |  | -0.224 | p<0.001 | -0.260 | -0.188 |
| Sex |  | 0.233 | p<0.001 | 0.208 | 0.258 |
| Age |  | -0.020 | p<0.001 | -0.022 | -0.018 |
| *APOE* |  | -0.019 | 0.198 | -0.047 | 0.010 |
| Cardiovascular problems |  | -0.076 | p<0.001 | -0.105 | -0.048 |
| Ethnicity |  | -0.364 | p<0.001 | -0.422 | -0.306 |
| TDI |  | -0.035 | p<0.001 | -0.040 | -0.031 |

Supplementary Table 1

Associations between inflammatory biomarker score quartiles and baseline cognitive tasks adjusted for age, sex*, APOE* ε4 status, cardiovascular problems, ethnic background and Townsend Deprivation Index (TDI).
