# Supplementary material for: Association between an inflammatory biomarker score and future dementia diagnosis in the population-based UK Biobank cohort of 500,000 people
Source: PLoS One. 2023 Jul 19;18(7):e0288045. doi: 10.1371/journal.pone.0288045 (PMC10355406; doi:10.1371/journal.pone.0288045)
Supplement: S2 Table — (DOCX) [file pone.0288045.s002.docx]

| Predictors | OR | Coefficient | p-value | 95% CI lower | 95% CI upper |
| --- | --- | --- | --- | --- | --- |
| **Prospective memory (UKB Field Code 20018)** | | | | | |
| *APOE ε4* non-carriers | n=103,038 |  |  |  |  |
| 1st quartile | Reference |  |  |  |  |
| 2nd quartile | 1.087 |  | p<0.001 | 1.039 | 1.137 |
| 3rd quartile | 1.099 |  | p<0.001 | 1.051 | 1.149 |
| 4th quartile | 1.212 |  | p<0.001 | 1.160 | 1.267 |
| Sex | 0.916 |  | p<0.001 | 0.889 | 0.945 |
| Age | 1.041 |  | p<0.001 | 1.039 | 1.043 |
| Cardiovascular problems | 1.110 |  | p<0.001 | 1.073 | 1.147 |
| Ethnicity | 2.607 |  | p<0.001 | 2.504 | 2.714 |
| TDI | 1.066 |  | p<0.001 | 1.061 | 1.072 |
| *APOE ε4* carriers | n=37,094 |  |  |  |  |
| 1st quartile | Reference |  |  |  |  |
| 2nd quartile | 1.050 |  | 0.182 | 0.977 | 1.128 |
| 3rd quartile | 1.123 |  | 0.001 | 1.045 | 1.206 |
| 4th quartile | 1.179 |  | p<0.001 | 1.096 | 1.269 |
| Sex | 0.925 |  | 0.003 | 0.879 | 0.974 |
| Age | 1.046 |  | p<0.001 | 1.043 | 1.050 |
| Cardiovascular problems | 1.095 |  | 0.001 | 1.035 | 1.157 |
| Ethnicity | 2.359 |  | p<0.001 | 2.225 | 2.502 |
| TDI | 1.067 |  | p<0.001 | 1.058 | 1.076 |
| **Verbal and numerical reasoning (Fluid intelligence, UKB Field Code 20016)** | | | | | |
| *APOE ε4* non-carriers | n=99,985 |  |  |  |  |
| 1st quartile | Reference |  |  |  |  |
| 2nd quartile |  | -0.123 | p<0.001 | -0.161 | -0.085 |
| 3rd quartile |  | -0.223 | p<0.001 | -0.261 | -0.186 |
| 4th quartile |  | -0.359 | p<0.001 | -0.397 | -0.322 |
| Sex |  | 0.242 | p<0.001 | 0.216 | 0.268 |
| Age |  | -0.019 | p<0.001 | -0.021 | -0.017 |
| Cardiovascular problems |  | -0.195 | p<0.001 | -0.225 | -0.166 |
| Ethnicity |  | -1.147 | p<0.001 | -1.189 | -1.105 |
| TDI |  | -0.074 | p<0.001 | -0.079 | -0.069 |
| *APOE ε4* carriers | n=36,024 |  |  |  |  |
| 1st quartile | Reference |  |  |  |  |
| 2nd quartile |  | -0.129 | p<0.001 | -0.188 | -0.069 |
| 3rd quartile |  | -0.244 | p<0.001 | -0.304 | -0.184 |
| 4th quartile |  | -0.386 | p<0.001 | -0.448 | -0.323 |
| Sex |  | 0.258 | p<0.001 | 0.214 | 0.301 |
| Age |  | -0.024 | p<0.001 | -0.027 | -0.022 |
| Cardiovascular problems |  | -0.184 | p<0.001 | -0.233 | -0.135 |
| Ethnicity |  | -1.075 | p<0.001 | -1.136 | -1.014 |
| TDI |  | -0.074 | p<0.001 | -0.082 | -0.067 |
| **Processing speed (Reaction time, UKB Field Code 20023)** | | | | | |
| *APOE ε4* non-carriers | n=305,552 |  |  |  |  |
| 1st quartile | Reference |  |  |  |  |
| 2nd quartile |  | 2.782 | p<0.001 | 1.664 | 3.901 |
| 3rd quartile |  | 4.620 | p<0.001 | 3.503 | 5.736 |
| 4th quartile |  | 8.899 | p<0.001 | 7.781 | 10.017 |
| Sex |  | -19.437 | p<0.001 | -20.223 | -18.651 |
| Age |  | 4.193 | p<0.001 | 4.143 | 4.244 |
| Cardiovascular problems |  | 5.571 | p<0.001 | 4.673 | 6.470 |
| Ethnicity |  | 46.591 | p<0.001 | 45.028 | 48.153 |
| TDI |  | 2.709 | p<0.001 | 2.578 | 2.841 |
| *APOE ε4* carriers | n=111,804 |  |  |  |  |
| 1st quartile | Reference |  |  |  |  |
| 2nd quartile |  | 1.895 | 0.035 | 0.132 | 3.659 |
| 3rd quartile |  | 3.182 | 0.001 | 1.384 | 4.981 |
| 4th quartile |  | 8.670 | p<0.001 | 6.799 | 10.542 |
| Sex |  | -17.448 | p<0.001 | -18.746 | -16.149 |
| Age |  | 4.183 | p<0.001 | 4.099 | 4.266 |
| Cardiovascular problems |  | 5.035 | p<0.001 | 3.559 | 6.512 |
| Ethnicity |  | 43.672 | p<0.001 | 41.413 | 45.931 |
| TDI |  | 2.973 | p<0.001 | 2.756 | 3.190 |
| **Visual declarative memory (Pairs matching, UKB Field Code 399)** | | | | | |
| *APOE ε4* non-carriers | n=308,086 |  |  |  |  |
| 1st quartile | Reference |  |  |  |  |
| 2nd quartile |  | -0.031 | 0.076 | -0.064 | 0.003 |
| 3rd quartile |  | -0.045 | 0.008 | -0.079 | -0.012 |
| 4th quartile |  | -0.096 | p<0.001 | -0.129 | -0.062 |
| Sex |  | -0.051 | p<0.001 | -0.074 | -0.027 |
| Age |  | 0.060 | p<0.001 | 0.058 | 0.061 |
| Cardiovascular problems |  | 0.045 | 0.001 | 0.017 | 0.072 |
| Ethnicity |  | 0.786 | p<0.001 | 0.740 | 0.832 |
| TDI |  | 0.025 | p<0.001 | 0.021 | 0.029 |
| *APOE ε4* carriers | n=112,741 |  |  |  |  |
| 1st quartile | Reference |  |  |  |  |
| 2nd quartile |  | -0.036 | 0.179 | -0.090 | 0.017 |
| 3rd quartile |  | -0.083 | 0.003 | -0.137 | -0.028 |
| 4th quartile |  | -0.097 | 0.001 | -0.153 | -0.040 |
| Sex |  | -0.040 | 0.045 | -0.079 | -0.001 |
| Age |  | 0.061 | p<0.001 | 0.058 | 0.063 |
| Cardiovascular problems |  | 0.027 | 0.227 | -0.017 | 0.072 |
| Ethnicity |  | 0.699 | p<0.001 | 0.633 | 0.766 |
| TDI |  | 0.028 | p<0.001 | 0.021 | 0.034 |
| **Working memory (Numeric memory, UKB Field Code 4282)** | | | | | |
| *APOE ε4* non-carriers | n=31,404 |  |  |  |  |
| 1st quartile | Reference |  |  |  |  |
| 2nd quartile |  | -0.087 | p<0.001 | -0.129 | -0.045 |
| 3rd quartile |  | -0.112 | p<0.001 | -0.154 | -0.070 |
| 4th quartile |  | -0.246 | p<0.001 | -0.288 | -0.204 |
| Sex |  | 0.222 | p<0.001 | 0.193 | 0.251 |
| Age |  | -0.019 | p<0.001 | -0.021 | -0.017 |
| Cardiovascular problems |  | -0.072 | p<0.001 | -0.106 | -0.038 |
| Ethnicity |  | -0.387 | p<0.001 | -0.456 | -0.319 |
| TDI |  | -0.037 | p<0.001 | -0.042 | -0.031 |
| *APOE ε4* carriers | n=11,182 |  |  |  |  |
| 1st quartile | Reference |  |  |  |  |
| 2nd quartile |  | 0.000 | 0.997 | -0.066 | 0.067 |
| 3rd quartile |  | -0.089 | 0.009 | -0.156 | -0.022 |
| 4th quartile |  | -0.162 | p<0.001 | -0.234 | -0.091 |
| Sex |  | 0.265 | p<0.001 | 0.216 | 0.314 |
| Age |  | -0.022 | p<0.001 | -0.025 | -0.019 |
| Cardiovascular problems |  | -0.085 | 0.003 | -0.140 | -0.030 |
| Ethnicity |  | -0.307 | p<0.001 | -0.416 | -0.198 |
| TDI |  | -0.031 | p<0.001 | -0.040 | -0.022 |

Supplementary Table 2

Associations between inflammatory biomarker score quartiles and baseline cognitive tasks adjusted for age, sex*, APOE* ε4 status, cardiovascular problems, ethnic background and Townsend Deprivation Index (TDI), the APOE ε4 non-carrier and carrier subsamples are analysed separately.
