# Supplementary material for: Association between an inflammatory biomarker score and future dementia diagnosis in the population-based UK Biobank cohort of 500,000 people
Source: PLoS One. 2023 Jul 19;18(7):e0288045. doi: 10.1371/journal.pone.0288045 (PMC10355406; doi:10.1371/journal.pone.0288045)
Supplement: S3 Table — (DOCX) [file pone.0288045.s003.docx]

|  |  |  |  |  |  |
| --- | --- | --- | --- | --- | --- |
|  |  |  |  |  |  |
| Predictors | OR | Coefficient | p-value | 95% CI lower | 95% CI upper |
| **Prospective memory (UKB Field Code 20018)** | | | | | |
| CRP only |  |  |  |  |  |
| 1st quartile | Reference |  |  |  |  |
| 2nd quartile | 1.033 |  | 0.086 | 0.995 | 1.072 |
| 3rd quartile | 1.097 |  | p<0.001 | 1.058 | 1.138 |
| 4th quartile | 1.146 |  | p<0.001 | 1.105 | 1.189 |
| Sex | 0.923 |  | p<0.001 | 0.900 | 0.947 |
| Age | 1.042 |  | p<0.001 | 1.041 | 1.044 |
| *APOE* | 0.983 |  | 0.259 | 0.955 | 1.012 |
| Cardiovascular problems | 1.113 |  | p<0.001 | 1.082 | 1.145 |
| Ethnicity | 2.481 |  | p<0.001 | 2.402 | 2.563 |
| TDI | 1.067 |  | p<0.001 | 1.063 | 1.072 |
| WBC only |  |  |  |  |  |
| 1st quartile | Reference |  |  |  |  |
| 2nd quartile | 1.028 |  | 0.142 | 0.991 | 1.067 |
| 3rd quartile | 1.087 |  | p<0.001 | 1.048 | 1.128 |
| 4th quartile | 1.153 |  | p<0.001 | 1.112 | 1.195 |
| Sex | 0.922 |  | p<0.001 | 0.899 | 0.945 |
| Age | 1.043 |  | p<0.001 | 1.041 | 1.044 |
| *APOE* | 0.979 |  | 0.14 | 0.951 | 1.007 |
| Cardiovascular problems | 1.119 |  | p<0.001 | 1.088 | 1.150 |
| Ethnicity | 2.528 |  | p<0.001 | 2.448 | 2.610 |
| TDI | 1.067 |  | p<0.001 | 1.062 | 1.071 |
| **Verbal and numerical reasoning (Fluid intelligence, UKB Field Code 20016)** | | | | | |
| 1st quartile | Reference |  |  |  |  |
| 2nd quartile |  | -0.091 | p<0.001 | -0.122 | -0.060 |
| 3rd quartile |  | -0.193 | p<0.001 | -0.224 | -0.162 |
| 4th quartile |  | -0.287 | p<0.001 | -0.319 | -0.256 |
| Sex |  | 0.242 | p<0.001 | 0.220 | 0.264 |
| Age |  | -0.020 | p<0.001 | -0.021 | -0.018 |
| *APOE* |  | -0.012 | 0.348 | -0.037 | 0.013 |
| Cardiovascular problems |  | -0.204 | p<0.001 | -0.229 | -0.179 |
| Ethnicity |  | -1.095 | p<0.001 | -1.129 | -1.061 |
| TDI |  | -0.075 | p<0.001 | -0.079 | -0.071 |
| WBC only |  |  |  |  |  |
| 1st quartile | Reference |  |  |  |  |
| 2nd quartile |  | -0.087 | p<0.001 | -0.118 | -0.056 |
| 3rd quartile |  | -0.197 | p<0.001 | -0.228 | -0.166 |
| 4th quartile |  | -0.300 | p<0.001 | -0.331 | -0.269 |
| Sex |  | 0.250 | p<0.001 | 0.228 | 0.272 |
| Age |  | -0.021 | p<0.001 | -0.023 | -0.020 |
| *APOE* |  | 0.018 | 0.144 | -0.006 | 0.042 |
| Cardiovascular problems |  | -0.199 | p<0.001 | -0.223 | -0.174 |
| Ethnicity |  | -1.131 | p<0.001 | -1.164 | -1.097 |
| TDI |  | -0.075 | p<0.001 | -0.079 | -0.072 |
| **Processing speed (Reaction time, UKB Field Code 20023)** | | | | | |
| CRP only |  |  |  |  |  |
| 1st quartile | Reference |  |  |  |  |
| 2nd quartile |  | 0.545 | 0.254 | -0.392 | 1.483 |
| 3rd quartile |  | 1.855 | p<0.001 | 0.910 | 2.799 |
| 4th quartile |  | 5.616 | p<0.001 | 4.662 | 6.569 |
| Sex |  | -18.816 | p<0.001 | -19.484 | -18.149 |
| Age |  | 4.185 | p<0.001 | 4.142 | 4.228 |
| *APOE* |  | 0.856 | 0.026 | 0.104 | 1.607 |
| Cardiovascular problems |  | 5.854 | p<0.001 | 5.096 | 6.613 |
| Ethnicity |  | 45.267 | p<0.001 | 44.003 | 46.531 |
| TDI |  | 2.818 | p<0.001 | 2.707 | 2.930 |
| WBC only |  |  |  |  |  |
| 1st quartile | Reference |  |  |  |  |
| 2nd quartile |  | 2.169 | p<0.001 | 1.249 | 3.088 |
| 3rd quartile |  | 4.079 | p<0.001 | 3.156 | 5.003 |
| 4th quartile |  | 7.881 | p<0.001 | 6.952 | 8.811 |
| Sex |  | -19.094 | p<0.001 | -19.750 | -18.439 |
| Age |  | 4.211 | p<0.001 | 4.169 | 4.253 |
| *APOE* |  | 0.420 | 0.263 | -0.315 | 1.155 |
| Cardiovascular problems |  | 5.875 | p<0.001 | 5.129 | 6.620 |
| Ethnicity |  | 45.943 | p<0.001 | 44.696 | 47.189 |
| TDI |  | 2.831 | p<0.001 | 2.721 | 2.940 |
| **Visual declarative memory (Pairs matching, UKB Field Code 399)** | | | | | |
| CRP only |  |  |  |  |  |
| 1st quartile | Reference |  |  |  |  |
| 2nd quartile |  | -0.073 | p<0.001 | -0.101 | -0.044 |
| 3rd quartile |  | -0.105 | p<0.001 | -0.133 | -0.076 |
| 4th quartile |  | -0.140 | p<0.001 | -0.169 | -0.112 |
| Sex |  | -0.053 | p<0.001 | -0.073 | -0.033 |
| Age |  | 0.060 | p<0.001 | 0.059 | 0.062 |
| *APOE* |  | 0.017 | 0.149 | -0.006 | 0.039 |
| Cardiovascular problems |  | 0.042 | p<0.001 | 0.019 | 0.065 |
| Ethnicity |  | 0.758 | p<0.001 | 0.721 | 0.795 |
| TDI |  | 0.026 | p<0.001 | 0.022 | 0.029 |
| WBC only |  |  |  |  |  |
| 1st quartile | Reference |  |  |  |  |
| 2nd quartile |  | -0.003 | 0.82 | -0.031 | 0.024 |
| 3rd quartile |  | -0.023 | 0.109 | -0.051 | 0.005 |
| 4th quartile |  | -0.052 | p<0.001 | -0.080 | -0.024 |
| Sex |  | -0.044 | p<0.001 | -0.064 | -0.025 |
| Age |  | 0.059 | p<0.001 | 0.058 | 0.061 |
| *APOE* |  | 0.029 | 0.01 | 0.007 | 0.051 |
| Cardiovascular problems |  | 0.037 | 0.001 | 0.015 | 0.059 |
| Ethnicity |  | 0.766 | p<0.001 | 0.729 | 0.803 |
| TDI |  | 0.024 | p<0.001 | 0.021 | 0.028 |
| **Working memory (Numeric memory, UKB Field Code 4282)** | | | | | |
| CRP only |  |  |  |  |  |
| 1st quartile | Reference |  |  |  |  |
| 2nd quartile |  | -0.045 | 0.011 | -0.079 | -0.010 |
| 3rd quartile |  | -0.100 | p<0.001 | -0.135 | -0.065 |
| 4th quartile |  | -0.196 | p<0.001 | -0.231 | -0.160 |
| Sex |  | 0.232 | p<0.001 | 0.207 | 0.257 |
| Age |  | -0.020 | p<0.001 | -0.021 | -0.018 |
| *APOE* |  | -0.024 | 0.089 | -0.052 | 0.004 |
| Cardiovascular problems |  | -0.081 | p<0.001 | -0.109 | -0.053 |
| Ethnicity |  | -0.357 | p<0.001 | -0.414 | -0.301 |
| TDI |  | -0.036 | p<0.001 | -0.040 | -0.031 |
| WBC only |  |  |  |  |  |
| 1st quartile | Reference |  |  |  |  |
| 2nd quartile |  | -0.024 | 0.176 | -0.059 | 0.011 |
| 3rd quartile |  | -0.091 | p<0.001 | -0.126 | -0.056 |
| 4th quartile |  | -0.155 | p<0.001 | -0.190 | -0.120 |
| Sex |  | 0.243 | p<0.001 | 0.218 | 0.267 |
| Age |  | -0.020 | p<0.001 | -0.022 | -0.019 |
| *APOE* |  | -0.007 | 0.62 | -0.035 | 0.021 |
| Cardiovascular problems |  | -0.089 | p<0.001 | -0.117 | -0.061 |
| Ethnicity |  | -0.370 | p<0.001 | -0.426 | -0.314 |
| TDI |  | -0.036 | p<0.001 | -0.040 | -0.031 |

Supplementary Table 3

Associations between inflammatory biomarker score quartiles separately for CRP and WBC and baseline cognitive tasks adjusted for age, sex*, APOE* ε4 status, cardiovascular problems, ethnic background and Townsend Deprivation Index (TDI).

CRP=C-reactive Protein, WBC=White Blood Cell Count
