# Supplementary material for: Association between an inflammatory biomarker score and future dementia diagnosis in the population-based UK Biobank cohort of 500,000 people
Source: PLoS One. 2023 Jul 19;18(7):e0288045. doi: 10.1371/journal.pone.0288045 (PMC10355406; doi:10.1371/journal.pone.0288045)
Supplement: S4 Table — (DOCX) [file pone.0288045.s004.docx]

| **Prospective memory (UKB Field Code 20018)** | |  |  |  |  |
| --- | --- | --- | --- | --- | --- |
| Predictors | OR | Coefficient | p-value | 95% CI lower | 95% CI upper |
| 1st quartile | Reference |  |  |  |  |
| 2nd quartile | 1.073 |  | p<0.001 | 1.032 | 1.115 |
| 3rd quartile | 1.098 |  | p<0.001 | 1.058 | 1.141 |
| 4th quartile | 1.196 |  | p<0.001 | 1.151 | 1.241 |
| Sex | 0.914 |  | p<0.001 | 0.890 | 0.938 |
| Age squared | 1.001 |  | p<0.001 | 1.001 | 1.002 |
| Age | 0.895 |  | p<0.001 | 0.875 | 0.917 |
| *APOE* | 0.979 |  | 0.152 | 0.950 | 1.008 |
| Cardiovascular problems | 1.105 |  | p<0.001 | 1.073 | 1.137 |
| Ethnicity | 2.502 |  | p<0.001 | 2.420 | 2.586 |
| TDI | 1.066 |  | p<0.001 | 1.061 | 1.071 |
| **Verbal and numerical reasoning (Fluid intelligence, UKB Field Code 20016)** | | |  |  |  |
| 1st quartile | Reference |  |  |  |  |
| 2nd quartile |  | -0.118 | p<0.001 | -0.150 | -0.086 |
| 3rd quartile |  | -0.219 | p<0.001 | -0.250 | -0.187 |
| 4th quartile |  | -0.353 | p<0.001 | -0.385 | -0.321 |
| Sex |  | 0.256 | p<0.001 | 0.234 | 0.278 |
| Age squared |  | -0.002 | p<0.001 | -0.002 | -0.002 |
| Age |  | 0.234 | p<0.001 | 0.215 | 0.254 |
| *APOE* |  | 0.002 | 0.881 | -0.023 | 0.027 |
| Cardiovascular problems |  | -0.188 | p<0.001 | -0.214 | -0.163 |
| Ethnicity |  | -1.117 | p<0.001 | -1.152 | -1.083 |
| TDI |  | -0.074 | p<0.001 | -0.077 | -0.070 |
| **Processing speed (Reaction time, UKB Field Code 20023)** | |  |  |  |  |
| 1st quartile | Reference |  |  |  |  |
| 2nd quartile |  | 2.478 | p<0.001 | 1.534 | 3.422 |
| 3rd quartile |  | 4.139 | p<0.001 | 3.191 | 5.088 |
| 4th quartile |  | 8.711 | p<0.001 | 7.752 | 9.670 |
| Sex |  | -18.971 | p<0.001 | -19.644 | -18.298 |
| Age squared |  | 0.019 | p<0.001 | 0.014 | 0.025 |
| Age |  | 2.041 | p<0.001 | 1.442 | 2.640 |
| *APOE* |  | 0.730 | 0.058 | -0.024 | 1.483 |
| Cardiovascular problems |  | 5.392 | p<0.001 | 4.624 | 6.159 |
| Ethnicity |  | 45.582 | p<0.001 | 44.297 | 46.867 |
| TDI |  | 2.773 | p<0.001 | 2.661 | 2.886 |
| **Visual declarative memory (Pairs matching, UKB Field Code 399)** | | |  |  |  |
| 1st quartile | Reference |  |  |  |  |
| 2nd quartile |  | -0.034 | 0.019 | -0.063 | -0.006 |
| 3rd quartile |  | -0.058 | p<0.001 | -0.087 | -0.030 |
| 4th quartile |  | -0.100 | p<0.001 | -0.129 | -0.071 |
| Sex |  | -0.050 | p<0.001 | -0.070 | -0.030 |
| Age squared |  | 0.001 | p<0.001 | 0.001 | 0.001 |
| Age |  | -0.014 | 0.13 | -0.032 | 0.004 |
| *APOE* |  | 0.028 | 0.014 | 0.006 | 0.051 |
| Cardiovascular problems |  | 0.039 | 0.001 | 0.016 | 0.062 |
| Ethnicity |  | 0.754 | p<0.001 | 0.717 | 0.792 |
| TDI |  | 0.025 | p<0.001 | 0.022 | 0.029 |
| **Working memory (Numeric memory, UKB Field Code 4282)** | |  |  |  |  |
| 1st quartile | Reference |  |  |  |  |
| 2nd quartile |  | -0.059 | 0.001 | -0.095 | -0.024 |
| 3rd quartile |  | -0.100 | p<0.001 | -0.135 | -0.064 |
| 4th quartile |  | -0.219 | p<0.001 | -0.255 | -0.183 |
| Sex |  | 0.236 | p<0.001 | 0.211 | 0.262 |
| Age squared |  | -0.001 | p<0.001 | -0.001 | -0.001 |
| Age |  | 0.066 | p<0.001 | 0.045 | 0.088 |
| *APOE* |  | -0.019 | 0.194 | -0.047 | 0.010 |
| Cardiovascular problems |  | -0.074 | p<0.001 | -0.103 | -0.046 |
| Ethnicity |  | -0.361 | p<0.001 | -0.419 | -0.303 |
| TDI |  | -0.035 | p<0.001 | -0.040 | -0.031 |

Supplementary Table 4

Associations between inflammatory biomarker score quartiles and baseline cognitive tasks adjusted for age squared, age, sex*, APOE* ε4 status, cardiovascular problems, ethnic background and Townsend Deprivation Index (TDI).
