# Supplementary material for: Association between an inflammatory biomarker score and future dementia diagnosis in the population-based UK Biobank cohort of 500,000 people
Source: PLoS One. 2023 Jul 19;18(7):e0288045. doi: 10.1371/journal.pone.0288045 (PMC10355406; doi:10.1371/journal.pone.0288045)
Supplement: S5 Table — (DOCX) [file pone.0288045.s005.docx]

| **Prospective memory (UKB Field Code 20018)** | | | | | |
| --- | --- | --- | --- | --- | --- |
| Predictors | OR | Coefficient | p-value | 95% CI lower | 95% CI upper |
| 1st quartile | Reference |  |  |  |  |
| 2nd quartile | 1.16 |  | 0.001 | 1.06 | 1.26 |
| 3rd quartile | 1.20 |  | p<0.001 | 1.10 | 1.32 |
| 4th quartile | 1.17 |  | 0.002 | 1.06 | 1.29 |
| Sex | 0.96 |  | 0.256 | 0.90 | 1.03 |
| Age | 1.06 |  | p<0.001 | 1.05 | 1.06 |
| *APOE* | 1.05 |  | 0.187 | 0.98 | 1.13 |
| Cardiovascular problems | 1.02 |  | 0.617 | 0.94 | 1.10 |
| Ethnicity | 2.58 |  | p<0.001 | 2.22 | 3.00 |
| TDI | 1.03 |  | p<0.001 | 1.02 | 1.05 |
| gap | 1.16 |  | p<0.001 | 1.14 | 1.19 |
| **Verbal and numerical reasoning (Fluid intelligence, UKB Field Code 20016)** | | | | | |
| 1st quartile | Reference |  |  |  |  |
| 2nd quartile |  | -0.140 | p<0.001 | -0.203 | -0.077 |
| 3rd quartile |  | -0.202 | p<0.001 | -0.268 | -0.136 |
| 4th quartile |  | -0.294 | p<0.001 | -0.365 | -0.222 |
| Sex |  | 0.313 | p<0.001 | 0.264 | 0.361 |
| Age |  | -0.024 | p<0.001 | -0.028 | -0.021 |
| *APOE* |  | -0.036 | 0.199 | -0.090 | 0.019 |
| Cardiovascular problems |  | -0.091 | 0.002 | -0.149 | -0.033 |
| Ethnicity |  | -1.117 | p<0.001 | -1.261 | -0.972 |
| TDI |  | -0.037 | p<0.001 | -0.046 | -0.028 |
| gap |  | -0.081 | p<0.001 | -0.096 | -0.067 |
| **Processing speed (Reaction time, UKB Field Code 20023)** | | | | | |
| 1st quartile | Reference |  |  |  |  |
| 2nd quartile |  | 1.676 | 0.299 | -1.488 | 4.840 |
| 3rd quartile |  | 3.798 | 0.025 | 0.483 | 7.112 |
| 4th quartile |  | 6.900 | p<0.001 | 3.315 | 10.486 |
| Sex |  | -20.742 | p<0.001 | -23.175 | -18.309 |
| Age |  | 4.324 | p<0.001 | 4.157 | 4.492 |
| *APOE* |  | 3.516 | 0.012 | 0.774 | 6.259 |
| Cardiovascular problems |  | -0.317 | 0.831 | -3.232 | 2.599 |
| Ethnicity |  | 27.284 | p<0.001 | 20.113 | 34.455 |
| TDI |  | 0.975 | p<0.001 | 0.516 | 1.434 |
| gap |  | 1.052 | 0.005 | 0.314 | 1.791 |
| **Visual declarative memory (Pairs matching, UKB Field Code 399)** | | | | | |
| 1st quartile | Reference |  |  |  |  |
| 2nd quartile |  | 0.038 | 0.403 | -0.051 | 0.126 |
| 3rd quartile |  | -0.036 | 0.451 | -0.128 | 0.057 |
| 4th quartile |  | -0.037 | 0.47 | -0.137 | 0.063 |
| Sex |  | 0.068 | 0.051 | 0.000 | 0.135 |
| Age |  | 0.055 | p<0.001 | 0.051 | 0.060 |
| *APOE* |  | -0.005 | 0.901 | -0.081 | 0.072 |
| Cardiovascular problems |  | 0.082 | 0.049 | 0.000 | 0.163 |
| Ethnicity |  | 0.723 | p<0.001 | 0.524 | 0.923 |
| TDI |  | 0.019 | 0.003 | 0.006 | 0.032 |
| gap |  | 0.052 | p<0.001 | 0.032 | 0.073 |
| **Working memory (Numeric memory, UKB Field Code 4282)** | | | | | |
| 1st quartile | Reference |  |  |  |  |
| 2nd quartile |  | -0.067 | 0.005 | -0.114 | -0.020 |
| 3rd quartile |  | -0.114 | p<0.001 | -0.164 | -0.064 |
| 4th quartile |  | -0.110 | p<0.001 | -0.165 | -0.056 |
| Sex |  | 0.271 | p<0.001 | 0.235 | 0.308 |
| Age |  | -0.024 | p<0.001 | -0.027 | -0.022 |
| *APOE* |  | -0.045 | 0.032 | -0.086 | -0.004 |
| Cardiovascular problems |  | -0.108 | p<0.001 | -0.153 | -0.062 |
| Ethnicity |  | -0.340 | p<0.001 | -0.452 | -0.228 |
| TDI |  | -0.013 | p<0.001 | -0.020 | -0.006 |
| gap |  | 0.006 | 0.471 | -0.010 | 0.022 |

Supplementary Table 5

Associations between inflammatory biomarker score quartiles and Instance 2 cognitive tasks adjusted for age, sex*, APOE* ε4 status, cardiovascular problems, ethnic background and Townsend Deprivation Index (TDI).

Gap=years elapsed between baseline and Instance 2.
