# Supplementary material for: Association between an inflammatory biomarker score and future dementia diagnosis in the population-based UK Biobank cohort of 500,000 people
Source: PLoS One. 2023 Jul 19;18(7):e0288045. doi: 10.1371/journal.pone.0288045 (PMC10355406; doi:10.1371/journal.pone.0288045)
Supplement: S6 Table — (DOCX) [file pone.0288045.s006.docx]

| *ε4* non-carriers subsample | n=308,086 |  |  |  |
| --- | --- | --- | --- | --- |
| Predictors | HR | p-value | 95% CI lower | 95% CI upper |
| 1st quartile | Reference |  |  |  |
| 2nd quartile | 1.261 | 0.004 | 1.075 | 1.478 |
| 3rd quartile | 1.330 | p<0.001 | 1.138 | 1.554 |
| 4th quartile | 1.570 | p<0.001 | 1.351 | 1.824 |
| sex | 1.246 | p<0.001 | 1.127 | 1.377 |
| Cardiovascular problems | 1.827 | p<0.001 | 1.650 | 2.023 |
| Ethnicity | 1.058 | 0.535 | 0.886 | 1.264 |
| TDI | 1.043 | p<0.001 | 1.027 | 1.059 |
|  |  |  |  |  |
| *ε4* carriers subsample | n=112,741 |  |  |  |
| Predictors | HR | p-value | 95% CI lower | 95% CI upper |
| 1st quartile | Reference |  |  |  |
| 2nd quartile | 1.034 | 0.659 | 0.891 | 1.199 |
| 3rd quartile | 1.180 | 0.026 | 1.020 | 1.364 |
| 4th quartile | 1.156 | 0.056 | 0.996 | 1.342 |
| sex | 1.046 | 0.39 | 0.944 | 1.160 |
| Cardiovascular problems | 1.956 | p<0.001 | 1.762 | 2.172 |
| Ethnicity | 1.003 | 0.973 | 0.847 | 1.187 |
| TDI | 1.023 | 0.006 | 1.007 | 1.040 |

Supplementary Table 6

Cox regression results of dementia diagnosis with sex, cardiovascular problems at baseline, ethnicity and Townsend Deprivation Index (TDI), analysed separately in the APO *ε4* non-carriers and APO *ε4* carriers subsamples.
