# Supplementary material for: Association between an inflammatory biomarker score and future dementia diagnosis in the population-based UK Biobank cohort of 500,000 people
Source: PLoS One. 2023 Jul 19;18(7):e0288045. doi: 10.1371/journal.pone.0288045 (PMC10355406; doi:10.1371/journal.pone.0288045)
Supplement: S7 Table — (DOCX) [file pone.0288045.s007.docx]

| CRP only |  |  |  |  |
| --- | --- | --- | --- | --- |
| Predictors | HR | p-value | 95% CI lower | 95% CI upper |
| 1st quartile | Reference |  |  |  |
| 2nd quartile | 0.931 | 0.169 | 0.840 | 1.031 |
| 3rd quartile | 1.004 | 0.938 | 0.907 | 1.111 |
| 4th quartile | 1.148 | 0.006 | 1.040 | 1.268 |
| Sex | 1.159 | p<0.001 | 1.080 | 1.245 |
| *APOE* | 2.598 | p<0.001 | 2.419 | 2.790 |
| Cardiovascular problems | 1.934 | p<0.001 | 1.799 | 2.078 |
| Ethnicity | 0.993 | 0.905 | 0.880 | 1.119 |
| TDI | 1.036 | p<0.001 | 1.024 | 1.047 |
|  |  |  |  |  |
| WBC only |  |  |  |  |
| Predictors | HR | p-value | 95% CI lower | 95% CI upper |
| 1st quartile | Reference |  |  |  |
| 2nd quartile | 1.199 | 0.001 | 1.080 | 1.332 |
| 3rd quartile | 1.249 | p<0.001 | 1.126 | 1.386 |
| 4th quartile | 1.312 | p<0.001 | 1.185 | 1.454 |
| Sex | 1.143 | p<0.001 | 1.066 | 1.226 |
| *APOE* | 2.569 | p<0.001 | 2.396 | 2.755 |
| Cardiovascular problems | 1.911 | p<0.001 | 1.780 | 2.052 |
| Ethnicity | 1.026 | 0.672 | 0.911 | 1.156 |
| TDI | 1.034 | p<0.001 | 1.023 | 1.046 |

Supplementary Table 7

Cox regression results for separate inflammatory biomarker score quartiles for CRP and WBC and dementia risk adjusted for sex*, APOE* ε4 status, cardiovascular problems, ethnic background and Townsend Deprivation Index (TDI).

CRP=C-reactive Protein, WBC=White Blood Cell Count
