# Supplementary material for: Association between an inflammatory biomarker score and future dementia diagnosis in the population-based UK Biobank cohort of 500,000 people
Source: PLoS One. 2023 Jul 19;18(7):e0288045. doi: 10.1371/journal.pone.0288045 (PMC10355406; doi:10.1371/journal.pone.0288045)
Supplement: Attachment — Submitted filename: Response_to_Reviewers.docx [file pone.0288045.s008.docx]

PONE-D-22-33736

Association between an inflammatory biomarker score and future dementia diagnosis in the population-based UK Biobank cohort of 500,000 people

PLOS ONE

Rebuttal letter

***We would like to thank the Reviewers for carefully evaluating our manuscript. We thoroughly considered the recommendations and comments and decided to carry out each of the recommended analyses and modifications. We think that these are extremely insightful, and adding them to the manuscript increases its quality, helps interpret the results and adds new knowledge to the existing.***

***We kindly ask the Reviewers to re-evaluate our revised manuscript; we are very grateful for their work.***

Reviewer #1: The authors used data from the UK Biobank to examine the association of a composite of inflammatory biomarkers measured in blood with concurrent cognition (measured across 5 cognitive domains) and incident dementia risk over an 11-year follow-up period. In this sample of 474,233 participants, the authors found that elevated inflammatory biomarker level was associated with worse performance on measures of prospective memory, fluid intelligence, and reaction time. This is a great data set and the authors set out to answer an important question. With the large amount of information available through UKB, there are a number of steps the authors could take to improve their study design and the interpretability of their findings. I provide specific critiques below.

In the intro the authors discuss the inconsistent findings from studies looking at immune markers in AD and MCI individuals and state that these inconsistencies make it difficult to develop validated biomarkers of early diagnosis and disease. Two points that I think the authors should address in conjunction with this statement. 1) there have been several fairly recent meta-analyses on this topic which show fairly consistent associations for a number of inflammatory or anti-inflammatory proteins. These meta-analyses have nearly 200 studies in them. Simply saying the results are inconsistent without providing some summary of the many studies on this topic doesn’t help advance the readers understanding of a well established area of research. 2) immune biomarkers will likely never be used as true biomarkers for AD. They’re not specific to any one neurodegenerative etiology and will unlikely be able to outperform well validated measures such as amyloid-beta 42 and soluble ptau217 that are disease-specific and represent indicators of AD’s defining features. That said, I do however think it’s immensely important to study inflammatory proteins in blood and in CSF to understand more about the underlying disease biology and he role of immunity.
***We thank this Reviewer for raising this important point. We added two sentences about the recent meta-analyses in the second paragraph of the Introduction.***

*‘Searching for other mechanisms highlighted the potential role of neuroinflammation in the dementia pathology. Measuring proinflammatory and anti-inflammatory cytokines in the cerebrospinal fluids and plasma of AD and mild cognitive impairment patients in small studies with typically less than 100 cases gave controversial or inconclusive results [3,4]. However, recent large-scale meta-analyses support the hypothesis of immune dysregulation in AD and mild cognitive impairment, reporting elevated peripheral levels of CRP, IL6 and IL1β in AD compared to controls [5, 6]. Dividing dementia according to aetiology, compared to healthy controls a moderate to large elevation of both blood IL6 and TNFα levels was associated with vascular dementia diagnosis. Blood IL6 levels were significantly different between vascular dementia patients and AD patients, and higher IL6 levels were also associated with incident vascular dementia in a meta-analysis of 20 studies [7]. ‘*

***We also included sentences in the Discussion part to acknowledge the limits of using inflammatory biomarkers as dementia predictors.***

*‘Nevertheless, although inflammatory biomarkers are not specific for dementia and appear to have a small effect, they may be useful in identifying individuals at risk for inflammation-dysregulation diseases (such as frailty and dementia) possibly at early stage of the condition and might enhance the precision of dementia predicting models.’*

Inflammatory proteins have also been implicated – perhaps even more strongly – in vascular cognitive impairment and vascular dementia. This should be acknowledged in the introduction, especially that 1) the authors are examining all-cause dementia as an outcome, and 2) because of the high rates of cerebrovascular disease in patients with Alzheimer’s disease.

***We treated this point together with the previous one, such the modified text in the Introduction part reflects the findings from large-scale meta-analyses reporting consistent results on associations between dementia and inflammatory biomarkers, and vascular dementia and inflammation.(Please see text at the previous point).***

Some rationale for the reason to exclude participants with APOEe2/APOEe4 should be provided in the genetics data section.

***To clarify this, we modified the text in the Materials and Methods section.***

*‘Individuals with the APOE ε2/APOE ε4 (protective/risk) genotypes were excluded from the analysis.’*

Age, sex, and APOEe4 were the only covariates included in the author’s regression models. However, there are many other potential confounders known to both influence inflammation and cognition/dementia that should be considered. Race, education (or another SES proxy), as well as cardiovascular risk factors should be considered – perhaps in a different model – as additional covariates. Without this sort of adjustment, the findings and challenging to interpret.

***We thoroughly adopted this important suggestion and included the suggested covariates, i.e., ethnicity, cardiovascular problems and material deprivation, in the analysis.***

***We added the description of these variables in the Materials and Methods section and reported the results in the Results section as well as in the Supplementary material. As these covariates proved to be important, we removed the original analyses with only age, sex and APOE ε4 status and replaced them with results of a model with age, sex, APOE ε4 status, ethnicity, presence of cardiovascular problems and material deprivations as covariates.***

Related to the comment above, Table 1 should be expanded to provide the reader with more information about the study sample. Things like race, education, APOEe4 status, clinical diagnoses (hypertension, diabetes) should be included. I also suggest splitting Table 1 by inflammatory biomarker quartile rather than by control vs. dementia, which is less informative.

***We thank for the Reviewer for this suggestion. We removed the original table (retaining its information in the text) and replaced it with an expanded Table 1, containing the suggested descriptive measures. The new table is split by biomarker quartiles rather than dementia diagnosis, as suggested.***

***This point was also made by Reviewer 3; therefore the new table accommodates the suggestions made by him/her as well.***

Was time-to-event data available to look at dementia risk using Cox models. I imagine this would be a more powerful way of detecting effects. The fact that people with dementia within 3 years of the baseline visit were excluded suggests that the date of dementia onset can be determined.

***Indeed, dementia onset data was available, therefore we performed Cox regression and presented the results in the relevant sections. We performed Cox regression for the original analysis (replacing the logistic regression) and also separately for CPR and WBC, as well as for APOE ε4 non-carriers and carriers. Finally, Cox models were applied for the separate AD and vascular dementia analyses.***

***This point was raised by Reviewer 3, too.***

The authors are encouraged to look at dementia risk by etiology. The relationship between inflammatory composite score and Alzheimer’s dementia and vascular dementia should be examined.
***This analysis was carried out and proved to be a very important point in understanding the role of inflammation in dementia. We added the results in a table format and discussed the results in the Discussion section.***

Because there are only 2 proteins in the composite score, it makes sense to look at them individually as well to see if either has an especially strong or especially weak association with the outcomes.

***We carried out the separate CRP and WBC association analyses for dementia and cognitive measures. The results highlighted the importance of classification of inflammation, not only dementia. We plan to follow up this in a subsequent study and thank for the Reviewer for raising this question.***

***We present the results in full in the Supplementary material.***

***This point was also raised by Reviewer 3.***

The authors have APOEe4 information. It would be informative to know whether the biomarker-cognition and biomarker-dementia relationships differed by APOEe4 possession status.

***This was another point of special importance, and we thank for the Reviewer for drawing our attention to it. The results are presented in the Results section as well as in the Supplementary material. Dividing the sample by APEO ε4 carrier status gave some insight into the mechanistic pathways, indicating that the role of inflammation in dementia might be different in non-carrier and carrier individuals.***

The authors should provide a rationale for adjusting for baseline cognitive variables in their examination of proteins in relation to dementia risk.

***After some consideration this analysis was removed from the manuscript as did not provide useful information.***

Reviewer #2: This research article titled “Association between an inflammatory biomarker score and future dementia diagnosis in the
population-based UK Biobank cohort of 500,000 people” by Mekli et al. Peripheral inflammation is an important topic in the context of dementia prevention and has shown to be related to cognitive impairments. This research study investigated peripheral inflammation for cognitive impairment and dementia risk. Although it is somewhat confirmatory study, it provided important information from a huge real world cohort. There are only some comments from me:
1. The major limitation for the study was cross-sectional for cognitive impairment.

***Please see our response under the next point below.***

2. There was not enough discussion on why peripheral inflammation was associated with better performance of pair match. This was a cross-sectional finding, and it is unknown if they are still positively associated in a follow-up.

***We thank for this Reviewer for pointing this out, indeed cognitive performance/impairment can only be adequately investigated in longitudinal studies. We attempted to overcome this to a certain extent by using cognitive performance results from a later time. We performed the same analyses using the baseline inflammatory biomarker quartiles as predictors and the cognitive measures at Instance 2 (4-13 years later) as outcomes, adjusting for sex, age, APOE ε4 status, cardiovascular problems, ethnicity, material deprivation as before and additionally time lapsed between baseline and Instance 2 (in years). We present the results briefly in the Results section and fully in the Supplementary material. We found that associations observed at baseline still hold at least on a p=0.005 level, except for Reaction time, where only the highest quartile remained significantly associated to the outcome. For Pairs matching, none of the quartiles remained significantly associated. This task was and exception in all analyses for reasons unknown for us.***

***Similar question was asked about the Pair matching test by Reviewer 3 and following his/her recommendations we found little correlations between the Pairs matching test and other tasks or with itself in a later instance (results presented in the Results section).***

3. The time between blood tests and cognitive tests were variable for different participants and should be controlled in the analyses.

***We thank for the Reviewer for this insightful comment. It did appear to be a time difference and we developed a variable accounting for it. We performed the cognitive test analyses including this in the models. We present the results in the relevant section and note that although this variable was significantly associated to the outcome for each cognitive task, it did not change the results noticeably.***

Reviewer #3: The authors assessed the association between a composite inflammatory biomarker score and five cognitive scores and also the development of dementia in following 3-11 years using data from the UK biobank.

I have some questions about the data analysis.

In the regression models, was age entered as a continuous variable? The relationship with age may not be linear, a spline or other functions of age may be needed in the model. What about the interaction between age and sex? Is it associated with the outcomes?

***We thank for raising this point. Age was entered as a continuous variable in this study. We carried out the suggested analyses by adding the squared age and found that the relationship with age is curvilinear. We presented the results briefly in the Results section, and fully in the Supplementary material but did not discuss them further, because this finding is likely to be followed up in a subsequent study. We thank the Reviewer for pointing this out.***

*‘To model the possible accelerating decline pattern of the cognitive function, we added the age squared variable in the regression models together with the previous ones. Age squared was significant in each analysis (p<0.001) without much decrease in the effect sizes of the quartiles or in their significance levels. The significant association between the two variables and cognition showed that the trajectories of cognition in later life have curvilinear shapes. These results are presented with all the covariates in Supplementary Table 4.’*

***We also included an age and sex interaction term in the model and reported the results in the Results section as follows.***

*‘We have included the interaction between age and sex in the analysis and the results show significant associations between the interaction between age and sex and Reaction time (p<0.001), Fluid intelligence (p=0.015), Pairs matching (p=0.011) and Working memory (0.021). These findings suggest that the changes of the four measures of cognitive functions among males as they get older differ from females.’*

There seems to be a trend in the effects of the quartiles. What if the composite biomarker score is used as a continuous variable instead of quartiles?

***We performed this analysis and presented the association results in the Results section.***

A cox model is a more appropriate model for the incident dementia risk analysis instead of a logistic model. Age can be used as the primary time scale.

***Following the advice from Reviewer 1 and Reviewer 3, we performed Cox regression and presented the results in the relevant sections***.

How correlated are the five cognitive scores? It might help us to understand why the pair matching result seemed counterintuitive.

***The Pairs matching test was an exception in all analyses and the only one with APOE ε4 being significant at some level (p=0.015). This task showed little correlation with the other 4 tasks or with itself in a later instance (Instance 2, 4-13 years later) (results are presented in the Results section). To speculate on the reasons is beyond the scope of this manuscript but there might be some random element in the test, or an underlying factor we missed.***

***Reviewer 2 also queried the Pairs matching test results and following his/her suggestion, we performed association analyses between baseline biomarker quartiles and cognitive scores at Instance 2, 3-14 years later. Whilst the associations remained significant for the other tasks (except for Reaction time, where only the 4^th^ quartile), for the Pairs matching none of the quartiles remained significantly associated with the outcome (p>=0.4). Unfortunately, it does not clarify the issue.***

Have the authors analyzed the two components of the composite biomarker score separately to see if one may contribute to the association more?

***This question was also raised by Reviewer 1 and proved to be hugely insightful. Based on the results we decided to design a follow up study to investigate further. We would like to thank this Reviewer for highlighting this important question.***
***Because of their importance, we presented the results in full in the Supplementary material.***

Individuals outside 3 SD of the scores were not included in the analysis. What proportion of the samples were in this outlier group?

***We added the information to the Materials and Methods section.***

*‘We only used the combined z-scores within 3SD of study mean to exclude participants with acute inflammation status (n=6,466, 1.42%).’*

Table 1 should include descriptive statistics of more variables, such as APOE genotype, the five cognitive scores. Sample size should be included as well. Better to show row percentages instead of column percentages.

***This point was also made by Reviewer 1; therefore, we created a new Table 1, reflecting the suggestions made by both Reviewers.***

***We retained the information from the original Table in the text and show row percentages as suggested by this Reviewer.***

***On behalf of my Co-Authors, I would like to thank the Reviewers once again for their insightful comments and suggestions. I am very hopeful that the revised manuscript will meet with their expectations and will be decided to be suitable for publication.***

***Yours sincerely,***

***Dr Krisztina Mekli***
