# Supplementary material for: Association between an inflammatory biomarker score and future dementia diagnosis in the population-based UK Biobank cohort of 500,000 people
Source: PLoS One. 2023 Jul 19;18(7):e0288045. doi: 10.1371/journal.pone.0288045 (PMC10355406; doi:10.1371/journal.pone.0288045)
Supplement: Attachment — Submitted filename: Response_to_Reviewers.docx [file pone.0288045.s009.docx]

PONE-D-22-33736R1

Association between an inflammatory biomarker score and future dementia diagnosis in the population-based UK Biobank cohort of 500,000 people

PLOS ONE

Rebuttal letter

On behalf of my Co-Authors, I would like to thank the Reviewers for carefully evaluating the revised manuscript.

Reviewer 3 kindly provided us with more recommendations and a detailed guidance. As before, we are very grateful for these and took great care of them.

We would like to address the comments raised by Reviewer 3.

**Estimates from models stratified by APOE ε4 carrier status were reported. However, stratified analysis is not necessary if the effects are not different between APOE ε4 carriers vs non-carriers. A formal testing of the quartiles by carrier status can be performed and only results based on the combined data need to be shown if there are no interaction effects.**

*As this Reviewer has correctly noted, we did not find compelling evidence for interactions between inflammation biomarker quartiles and APOE ε4 status. Therefore, now we only mention these results briefly in the main text and pointing out the similarities and differences between the stratified and combined data analyses. All results have been moved to the Supplementary material.*

*This way we sought to give an overview for the Readers who can consult with the Supplementary material for more details.*

*We thank for the Reviewer for this suggestion.*

**A case-only approach can be used to evaluate if effects of inflammatory biomarker score were different for vascular dementia vs AD (ref: Wang et al Stat Med. 2016 Feb 28; 35(5): 782–800). The model will only include dementia cases (from both subtypes) and the response variable is a binary indicator variable for AD (or vascular dementia) status. A logistic model can be used to see if the inflammatory biomarker score is associated with dementia subtype while adjusting for other covariates. Under the null of no difference between the two subtypes there should be no association.**

*We thank for the Reviewer for this suggestion and specifically for giving us a very detailed guide on the correct way of performing the analysis. We performed the analysis as suggested using the Alzheimer’s disease category as reference. We present the results at the end of the Results section in text and Table 6.*

*We would like to thank for the Reviewer for the detailed guide on conducting the analysis as well as for the referenced paper.*

**This sentence: “The respondents were censored at the year dementia was first recorded or death, whichever came first.”- dementia is an event not a censoring.**

*We thank for the Reviewer for pointing out this error in the text. We corrected the text, now it reads:*

*‘The respondents were censored at the year of the last follow-up.’*

Once again, I would like to thank for the Reviewers for their contribution to improving the quality of the manuscript and recommending knowledge-enhancing analyses.

On behalf of my Co-Authors,

Krisztina Mekli

The University of Manchester
