# Supplementary material for: Association between an inflammatory biomarker score and future dementia diagnosis in the population-based UK Biobank cohort of 500,000 people
Source: PLoS One. 2023 Jul 19;18(7):e0288045. doi: 10.1371/journal.pone.0288045 (PMC10355406; doi:10.1371/journal.pone.0288045)
Supplement: Attachment — Submitted filename: Response_to_Reviewers.docx [file pone.0288045.s010.docx]

Response to Reviewers

We thank again for all the Reviewers for reading and correcting our *manuscript*, their excellent comments, and insightful recommendations. This time we would especially thank for Reviewer 3 for their help in interpreting correctly the results of the case-only analysis.

Reviewer #3: **This revision has addressed all previous comments. I have one additional comment- the following sentence in the abstract is not quite accurate: “This association was probably driven by the vascular dementia cases in the samples (HR=1.483, p=0.055)”. The case-only approach is to assess if there is disease subtype heterogeneity. The marginally significant p value suggests that the association with highest biomarker quartile probably varies between disease subtypes (vascular dementia and Alzheimer’s disease).**

*We removed the incorrect interpretation and added the following sentence:*

*‘A case-only analysis to assess disease subtype heterogeneity suggested probable differences in the association with the highest biomarker quartile between vascular dementia and Alzheimer disease subtypes (OR=1.483, p=0.055).’*

On behalf of my Co-Authors, I would like to express my gratitude to the Reviewers for their work on this *manuscript*. Thank you very much for the knowledge-enhancing experience.

Sincerely yours,

Krisztina Mekli
